# Antioxidant Properties of Ergosterol and Its Role in Yeast Resistance to Oxidation

**DOI:** 10.3390/antiox10071024

**Published:** 2021-06-25

**Authors:** Sebastien Dupont, Paul Fleurat-Lessard, Richtier Gonçalves Cruz, Céline Lafarge, Cédric Grangeteau, Fairouz Yahou, Patricia Gerbeau-Pissot, Odonírio Abrahão Júnior, Patrick Gervais, Françoise Simon-Plas, Philippe Cayot, Laurent Beney

**Affiliations:** 1 UMR Procédés Alimentaires et Microbiologiques, University Bourgogne Franche-Comté, AgroSup Dijon, PAM UMR A 02.102, 21000 Dijon, France; richtier@hotmail.com (R.G.C.); celine.lafarge@agrosupdijon.fr (C.L.); cedric.grangeteau@agrosupdijon.fr (C.G.); yahou.fairouz@gmail.com (F.Y.); gervais@u-bourgogne.fr (P.G.); philippe.cayot@agrosupdijon.fr (P.C.); 2 Institut de Chimie Moléculaire de l’Université de Bourgogne, University Bourgogne Franche-Comté, ICMUB—UMR CNRS 6302, CEDEX, 21078 Dijon, France; Paul.Fleurat-Lessard@u-bourgogne.fr; 3 Department Agroindústria, Alimentos e Nutrição, Escola Superior de Agricultura ‘Luiz de Queiroz’, University of São Paulo, Piracicaba 13418-900, Brazil; 4 UMR1347 Agroécologie, ERL 6300 CNRS, INRA, CEDEX, 21065 Dijon, France; Patricia.Gerbeau-Pissot@u-bourgogne.fr (P.G.-P.); francoise.simon-plas@inrae.fr (F.S.-P.); 5 Instituto de Ciências Biológicas e Naturais, Universidade Federal do Triangulo Mineiro, Uberaba 38025-180, Brazil; odonirio.junior@uftm.edu.br

**Keywords:** sterol, oxidation, antioxidant, lipids, yeast, plasma membrane

## Abstract

Although the functions and structural roles of sterols have been the subject of numerous studies, the reasons for the diversity of sterols in the different eukaryotic kingdoms remain unclear. It is thought that the specificity of sterols is linked to unidentified supplementary functions that could enable organisms to be better adapted to their environment. Ergosterol is accumulated by late branching fungi that encounter oxidative perturbations in their interfacial habitats. Here, we investigated the antioxidant properties of ergosterol using in vivo, in vitro, and in silico approaches. The results showed that ergosterol is involved in yeast resistance to tert-butyl hydroperoxide and protects lipids against oxidation in liposomes. A computational study based on quantum chemistry revealed that this protection could be related to its antioxidant properties operating through an electron transfer followed by a proton transfer mechanism. This study demonstrates the antioxidant role of ergosterol and proposes knowledge elements to explain the specific accumulation of this sterol in late branching fungi. Ergosterol, as a natural antioxidant molecule, could also play a role in the incompletely understood beneficial effects of some mushrooms on health.

## 1. Introduction

Since the discovery of cholesterol by Chevreul in the early nineteenth century, a huge diversity of sterols has been identified in the different kingdoms of eukaryotic cells [1]. Ergosterol, the main sterol of fungi, was first isolated from ergot in 1889 [2]. In the second half of the twentieth century, extensive research on sterol functions revealed that the sterols of the four eukaryotic kingdoms (Animalia, Plantae, Fungi, and Protista) are essential for the organization and function of cell plasma membranes [3]. However, the basis of the specificity of sterols in each of the kingdoms is unclear, although it could be related to a supplementary role of some sterols. 

Ergosterol is the typical sterol of late branching fungi, and is considered to be the equivalent of cholesterol in vertebrates and of phytosterols (the most representative being sitosterol, stigmasterol and campesterol) in plants. Ergosterol is essential to these fungi and its synthetic pathway is one of the main targets of antifungal strategies [4,5]. Even if many aspects of ergosterol biosynthesis regulation are still unknown, it involves a large number of *ERG* genes and the crosstalk between different environmental signals and pathways [6]. Ergosterol is produced from a biosynthetic pathway that has early steps in common with the cholesterol and phytosterol synthesis pathways, and involves final specific steps that lead to the formation of two conjugated double bonds in the B-ring of the sterol structure (Appendix A). This chemical characteristic is associated with an increased energy and metabolic cost compared with cholesterol and phytosterol synthesis [7]. However, the specificity of sterols in the different living kingdoms is more intricate. Weete and colleagues investigated the diversity of sterols in fungi and showed that ergosterol is the predominant sterol in the most evolved clades of fungi, while cholesterol and other sterols are found in the membranes of early branching fungi [8]. These findings suggest that the evolution of fungi has been accompanied by a tendency to accumulate ergosterol instead of cholesterol-related sterols. In a previous study, we showed that the final steps of ergosterol synthesis paralleled an increase in the resistance of the yeast *Saccharomyces cerevisiae* (ascomycota) to desiccation in air [9]. Therefore, we hypothesized that the ergosterol synthetic pathway appeared in late branching fungi during their transition from water to land. Indeed, in contrast to aquatic life, organisms living in interfacial habitats such as the surfaces of soil and rocks, or of living organisms (plants and animals)—particularly those that do not have the possibility of maintaining hydric homoeostasis or of moving to avoid sun injury—are exposed to unstable environmental conditions (temperature, moisture, and light). Some unicellular fungi exhibit remarkable resistance in these habitats, which constitute their main ecological niche. In particular, yeast is able to manage major hydric stress and resist prolonged periods of desiccation [10].

Cellular dehydration results in mechanical and structural constraints on cells, and leads to cell shrinkage, membrane deformation, aggregation of proteins and other contents, and phospholipid phase transitions [11,12,13]. Cell desiccation also promotes the formation of reactive oxygen species (ROS) by mechanisms related to metabolic arrest and enzyme dysfunction [14,15]. The promotion of oxidation in cells after drying is also directly related to the replacement of the surrounding water by air, because water attenuates light propagation and reduces the lifespan and velocity of ROS such as singlet oxygen more efficiently than air [16]. The cellular mechanisms of yeast adaptation to hydric fluctuations comprise constitutive and inducible processes involved in the management of oxidative and hydric stress. Part of the fungal arsenal to prevent desiccation damage is based on non-specific protective and repair tools also found in other eukaryotes, including catalase and superoxide dismutase. In late branching fungi, ergosterol is also involved in cell resistance to hydric perturbation. Its protective role has been reported to be related to its condensing effect on lipid bilayers, which contributes to the mechanical resistance of the plasma membrane [17]. However, it could also be based on antioxidant properties that prevent the peroxidation of phospholipids. In particular, based on similarities with the mechanism operating in tocopherol, the presence of two double bonds in the B-ring of ergosterol could confer antioxidant properties and thereby provide an adaptive advantage to late branching fungi in interfacial habitats. In a non-cellular context, it was observed that ergosterol protects yogurt from oxidation [18], but the mechanism involved in that effect was not studied. In another study, it was also observed that ergosterol is the main contributor to the antioxidant activity of the lipophilic fraction of button mushrooms [19].

The aim of the present study was to assess the antioxidant properties of ergosterol and to clarify the mechanisms behind the positive contribution of ergosterol to yeast resistance to oxidative treatment. In vitro and in silico approaches allowed us to demonstrate an antioxidant role of ergosterol, which protected the membrane lipids from oxidative perturbation. This property of ergosterol could be involved in the yeast resistance to oxidation. 

## 2. Materials and Methods

### 2.1. Yeast Strains and Growth Conditions

Four strains of *S. cerevisiae* yeast were used in this study. The wild-type strain BY4742 (*MAT*α *his3*Δ*1 leu2*Δ*0 lys2*Δ*0 ura3*Δ*0*) and the *erg6*Δ mutant (*MAT*α *his3*Δ*1 leu2*Δ*0 lys2Δ0::kanMX4*) were obtained from the EUROSCARF yeast-deletion library (EUROSCARF, Frankfurt, Germany). The wild-type strain RH448 (*MAT*a *leu2 ura3 his4 lys2 bar1*) and the *erg2*Δ*erg6*Δ mutant (*MAT*a *erg2(end11)-1*Δ*::URA3 erg6*Δ *leu2 ura3 bar1*) were kindly provided by H. Riezman of the University of Geneva [20]. Subculture was performed by introducing one colony of yeast into a 250 mL conical flask containing 100 mL of yeast extract–peptone–dextrose (YPD) medium, and was shaken at 250 rpm for 48 h at 25 °C on a rotary shaker (New Brunswick Scientific, Edison, NY, USA). Because of the slower growth of the double mutant, a subculture of the *erg2*Δ*erg6*Δ mutant was performed for 72 h. Then, 1 mL of subculture was transferred into a flask containing 100 mL of fresh YPD medium, and the cultures were shaken at 250 rpm for 24 h at 25 °C, and were allowed to grow to the early stationary phase. This time was increased to 48 h for the *erg2*Δ*erg6*Δ strain. A stationary phase of growth was chosen because yeasts are generally more resistant to various perturbations, including oxidative treatments, than they can be in the exponential phase [21,22]. The final population was estimated at about 10^8^ cells mL^−1^. Samples (20 mL) of cultures were centrifuged (5 min, 2800× *g*), washed twice in phosphate-buffered saline (PBS), and the cell concentration was adjusted to OD_600 nm_ = 0.5.

### 2.2. Sterol and Fatty Acid Analysis of the Different Strains of Yeast

For the total fatty acid analysis, transmethylation of fatty acids was performed for 1 h at 85 °C by mixing a yeast pellet (number of yeasts was assessed by flow cytometry for each pellet of the different strains of yeasts) with 1 mL of methanol:H_2_SO_4_ solution (100:2.5, *v*/*v*) containing the internal standards C17:0 (6.3 mg/mL). After cooling, 800 µL of hexane:2.5% NaCl (1:1, *v*/*v*) was added, and the upper hexane phase containing fatty acid methyl esters (FAMEs) was harvested and injected in a GC-FID chromatograph. GC-FID was performed using an Agilent 7890 gas chromatograph equipped with a DB-Wax column (15 m × 0.53 mm, 1 µm; Agilent, Santa Clara, CA, USA) and flame ionization detection. The temperature gradient was 160 °C for 1 min, increased to 190 °C at 20 °C/min, increased to 210 °C at 5 °C/min, and then remained at 210 °C for 5 min. The FAMEs were identified by comparing their retention times with the commercial fatty acid standards (Sigma-Aldrich, Saint Quentin Fallavier, France) and were quantified using ChemStation (Agilent) to calculate the peak surfaces, which were then compared with the C17:0 response.

For the sterol analysis, a saponification step was performed by adding 1 mL of ethanol and 0.1 mL of 11 N KOH with the internal standard a-cholestanol (50 µg/mL) to a yeast pellet and incubating it overnight at 80 °C. After the addition of 1 mL of hexane and 2 mL of water, the sterol-containing upper phase was recovered, and the solvent was evaporated under an N_2_ gas stream. The sterols were trimethylsilylated by N,O-bis(trimethylsilyl) trifluoroacetamide (BSTFA)-trimethylchlorosilane for 15 min at 115 °C. After complete evaporation of BSTFA under N_2_ gas, the derivatized sterols were dissolved in 0.4 mL of hexane and were analyzed by GC-MS. GC-MS was performed using an Agilent 6850 gas chromatograph and coupled MS detector MSD 5975-EI (Agilent). An HP-5MS capillary column (5% phenyl-methyl-siloxane, 30-m, 250-mm, and 0.25-mm film thickness; Agilent) was used with helium carrier gas at 2 mL/min; injection was done in splitless mode; the injector and mass spectrometry detector temperatures were set to 250 °C; and the oven temperature was held at 50 °C for 1 min, then programmed with a 25 °C/min ramp to 150 °C (2-min hold) and a 10 °C/min ramp to 320 °C (6-min hold).

### 2.3. Assessment of Oxidation Effects on Yeast Viability and Plasma Membrane Integrity

The oxidation effects on yeast physiology and viability were investigated after the induction of a free-radical reaction by tert-butyl hydroperoxide (t-BOOH). Yeasts were placed in PBS containing t-BOOH (4 or 6 mM) for 1 or 2 h. After these times, the cells were washed and their viability was assessed by the colony forming unit (CFU) method. Plasma membrane integrity was also measured by adding 2 μL of propidium iodide (PI) solution (1 mg/mL) to 1 mL of yeast (OD_600 nm_ = 0.5). PI crossed the plasma membrane of permeabilized cells and stained the nucleic acids. The proportion of stained cells was estimated by flow cytometry using a BD FACS Aria II Flow Cytometer (BD Biosciences, San José, CA, USA) equipped with a laser excitation line at 488 nm. PI fluorescence was detected at 610 nm with at least 10,000 cells included in each analysis.

### 2.4. Preparation of Liposomes from Natural Yeast Lipid Extracts

Liposomes were prepared from natural yeast polar lipid extracts of *S. cerevisiae* (Avanti Polar Lipids) using the film hydration method [23]. The phospholipids and the different sterols (zymosterol, cholesta-5,7,24-trienol (Avanti Polar Lipids), and ergosterol (Sigma)) were dissolved in chloroform. The organic solvent was evaporated to form a film that was dried under a stream of nitrogen for 1 h. This film was then hydrated with degassed PBS to obtain multilamellar vesicles. PBS was degassed of oxygen by nitrogen bubbling for 12 h. We prepared large unilamellar vesicles from the multilamellar ones using the extrusion technique. The suspension of multilamellar vesicles was transferred into a Liposofast small-volume extrusion device (Avestin Inc., Ottawa, ON, Canada) with a polycarbonate membrane with a 200 nm pore size, designed to obtain a homogeneous population of large unilamellar liposomes. The lipid concentration of the liposomes made only with yeast lipid extracts was 2.5 mM. For liposomes containing sterols, zymosterol, cholesta-5,7,24-trienol, or ergosterol was added to obtain a final concentration of 0.83 mM. This corresponds to a sterol/phospholipid molar ratio of 1/3. A control was also performed by adding tocopherol, a lipophilic antioxidant, in the same ratio of the one of sterols.

### 2.5. Assessment of Liposome Fluidity by Fluorescence Spectroscopy

The membrane fluidity of the different liposomes was assessed by steady-state fluorescence anisotropy of diphenyl-hexatriene (DPH). For that, 600 μL of the previously prepared liposome suspension was transferred to a quartz cuvette containing 2400 μL PBS. The liposomes were labelled with DPH (1 μL of 5 mM stock solution in tetrahydrofuran) in a dark at 25 °C for 5 min.

Steady-state anisotropy of DPH was measured in a Fluorolog-3 spectrometer (Horiba Jobin Yvon) using T-format fluorescence polarizers. During the measurements, samples were stirred and equilibrated in a temperature-controlled chamber using a thermoelectric Peltier junction. The excitation and emission wavelengths were 360 nm (5 nm bandwidth) and 431 nm (5 nm bandwidth), respectively. Steady-state fluorescence anisotropy (r) was calculated as follows:

$$r=\frac{I_{VV}-GI_{VH}}{I_{VV}+2GI_{VH}}$$

where *I* is the fluorescence intensity, and the first and second subscripts refer to the setting of the excitation and emission polarizers, respectively. $G=\frac{I_{HV}}{I_{HH}}$ is a correction factor for the monochromator’s transmission efficiency for vertically and horizontally polarized light.

### 2.6. Oxidation of Liposome Suspensions and Assessment of Lipid Peroxidation

The kinetics of lipid peroxidation were followed using the fluorescent probe C11-BODIPY 581/591 (C11-BP) at a final concentration of 20 μM. Liposomes were stained with the probe for 10 min before starting the oxidation treatments. Analyses were performed with a Fluorolog-3 spectrofluorimeter (Horiba Jobin Yvon). The excitation wavelength was set at 488 nm and the emission intensity ratio between the oxidized (520 nm) and the non-oxidized (591 nm) state of the probe was measured. During the measurements, the samples were stirred and equilibrated at 25 °C in a temperature-controlled chamber using a thermoelectric Peltier junction.

The oxidation of liposomes was induced by free-radical reactions. Lipid peroxidation was generated by the addition of 150 μL of 2 mM cumene hydroperoxide and 15 μL of 0.1 mM haemin. Emission spectra of C11-BP were acquired every 900 s and the I_520 nm/I590 nm_ ratio of C11-BP was calculated.

### 2.7. Assessment Hydrogen Atom-Donating Ability of Sterols by the DPPH Method

The DPPH method is based on the exchange of a proton and a single electron (radical), between the stable radical chromophore 2,2-diphenyl-1-picrylhydrazyl (DPPH^●^), and a molecule (A-H), generally an antioxidant; here, a sterol: DPPH^●^ + A-H → DPPH-H + A^●^. The radical DPPH^●^ absorbs at λ_max_ = 515 nm (E = 10,900 M^−1^·cm^−1^ in methanol) and gives a purple solution, generally in acetone, methanol, or ethanol (appropriate solvents of DPPH^●^). The non-radical DPPH-H resulting from the radical exchange with a molecule absorbs in the UV and gives a yellow solution. The observation of the decrease in optical density (OD) at 515 nm is usually carried out over a period of between 10 min [24] and a maximum of 300 min [25]. 

In our experiments, ergosterol (log *P* = 9.28) and zymosterol (log *P* = 9.32) were not soluble in ketone or alcohols, even in long-chain alcohols such as octanol and decanol, and the usual solvents for the DPPH^●^ method cannot be used to evaluate the potential ability of sterol to scavenge a radical. The sterols were easily dissolved in isooctane (2,2,4-trimethylpentane), a non-polar solvent; 7 mg of DPPH^●^ was dissolved in 100 mL of isooctane (1.7 × 10^−4^ M) to obtain an OD_515 nm_ around 1 after a 1:1 dilution. Because DPPH^●^ is poorly soluble in isooctane, an overnight dissolution in a screw-capped vial was necessary. Zymosterol and ergosterol solutions at different concentrations in isooctane were mixed 1/1 with the DPPH^●^ solution to obtain kinetics at molar ratios of sterol/DPPH^●^ from 0.1 to 5. The solutions of the DPPH^●^ and sterols were stored in a screw-capped vial until the OD_515 nm_ reached a stable value. Regular samplings were performed to follow the OD_515 nm_ during this time. Concerning statistical analysis, the experiments to obtain each point for the zymosterol at the different times were repeated three times, allowing us to calculate the 95% confidence intervals. For the ergosterol, the reaction kinetic was much faster and the different points were not obtained at the same time. We could not therefore calculate the confidence intervals for these points. So, we made the choice to show all of the obtained points (30) on the figure, in order to model them and to validate the modeling via a Pearson table.

The kinetics obtained at different ratios allowed us to calculate the EC_50_ and t_50_ values, corresponding to the respective effective concentrations of sterol, giving a final OD_515 nm_ of 50% of the initial OD_515 nm_ and the time required to reach the steady state with EC_50_. For each sterol/DPPH^●^ ratio, the OD_515 nm_ at infinity was determined from the graphic representations of OD_515 nm_ as a function of time. Then, the graphic representation of the OD_515 nm_ at infinity as a function of the different sterol/DPPH^●^ ratios was plotted. From this, the concentration required for an OD_515 nm_ at infinity of 0.5 was determined, corresponding to the EC_50_ value; t_50_ was then calculated. These two parameters allowed us to calculate the radical scavenging efficiency (RSE) as the inverse of the product of EC_50_ and t_50_ [26]: RSE = 1**/**(EC_50_ t_50_). The less time necessary to reach the end of the radical exchange, the lower the concentration of the molecule required to halve the initial OD_515 nm_ value (i.e., halve the initial concentration of radical DPPH^●^), and the higher the RSE value, the better the radical scavenger of sterol.

### 2.8. Computational Analysis of Antioxidant Properties of Sterols

The computational study was conducted on ergosterol, zymosterol, and cholesta-5,7,24-trienol. The structure of ergosterol in its bioactive conformation was obtained from the complex with an elicitin enzyme, specifically the secreted β-cryptogenin from *Phytophthora cryptogea*, whose crystallographic structure with the sterol was obtained in 1998 [27]. The structures of zymosterol and cholesta-5,7,24-trienol were obtained by modifying the structure obtained for ergosterol. The structures were optimized in solvent ethyl ethanoate, simulated from its dielectric constant of 5.9867 with the continuous polarized integral equation formalism polarizable continuum model (IEF-PCM model) [28]. Ethyl ethanoate was used as a solvent to mimic a biological lipid environment. The B3LYP DFT method with a 6-311+ G (2d,p) basis set was used in the Gaussian 16 program [29]. 

The radical cations were obtained by changing the charge and multiplicity in the input for unrestricted calculations of the oxidized molecules. The spin density of the oxidized molecules (minimized after the withdrawal of an electron) were plotted with ChemCraft [30] to evaluate the electronic delocalization of the unpaired electron. The electrostatic potential map was plotted using Gabedit [31] on the electronic density isosurface of 0.001, where blue indicates more positive regions and red the most negative regions. The final radical product was then obtained from the radical cations by deleting one H atom. Many positions were tried to subtract these hydrogen atoms—all of them are shown in Appendix A, while only the most stable ones are shown here for ergosterol, zymosterol, and cholesta-5,7,24-trienol.

## 3. Results

### 3.1. Sterol and Fatty Acid Composition of the Different Yeast Strains

**Figure 1:**
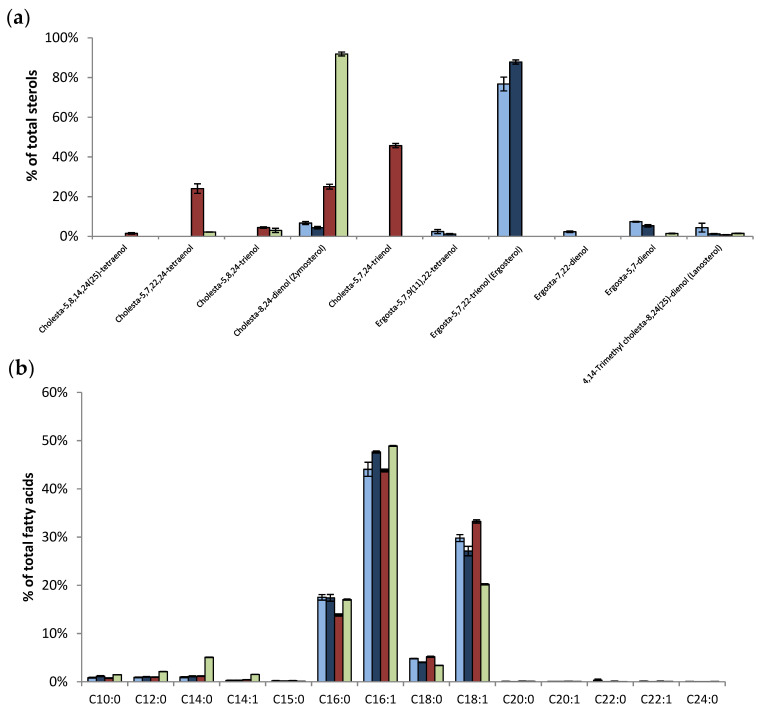
Lipid composition of the different *S. cerevisiae* strains. BY4742 WT (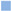), RH448 WT (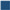), *erg6*Δ (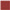), and *erg2*Δ*erg6*Δ (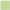) strains of *S. cerevisiae.* (**a**) Analysis of the sterol composition and the relative abundance. (**b**) Analysis of the fatty acid composition and the relative abundance. Vertical bars represent standard deviation.

Deletion of the genes involved in the ergosterol biosynthetic pathway has been described to strongly affect the nature of sterols accumulated by yeasts [32]. It exists only five viable mutants (cultivable without specific accommodations of a classical growth yeast medium) with single gene deletion in the ergosterol biosynthetic pathway (EBP): *erg6*Δ, *erg2*Δ, *erg3*Δ, *erg5*Δ, and *erg4*Δ. The successive action of the enzymes linked to these genes allows for the formation of ergosterol in wild type strains. In our study, the *erg6*Δ strain was chosen, because *erg6* is the first non-essential gene encoding for the enzyme that catalyzes the first of the five final steps of the EBP (See Appendix A). This strain accumulates mainly zymosterol, a sterol whose structure is very different from ergosterol, and cholesta-5,7,24-trienol, a sterol that exhibits two conjugated double bonds as ergosterol [20]. The *erg2*Δ*erg6*Δ double mutant was used in our study because it accumulates a large majority of zymosterol [20]. However, the sterol and fatty acid composition can be modified by growth conditions of yeasts. Sterols and fatty acids accumulated by the wild type and EBP mutant strains of our study were analyzed and are presented in Figure 1. 

Ergosterol was the main sterol accumulated by the two wild type strains of yeasts. Its proportion reached 77% and 88% for the BY4742 and RH448 strains, respectively (Figure 1a). The *erg2*Δ*erg6*Δ mutant accumulated mainly zymosterol (94%). Three main sterols were accumulated in the *erg6*Δ mutant, namely: 46% of cholesta-5,7,24-trienol, 25% of zymosterol, and 24% of cholesta-5,7,22,24-tetraenol. These proportions of sterols for the different strains were in agreement with previous studies of the literature. Concerning the fatty acid composition, the two wild type strains presented a similar profile with the following four main fatty acids: ≈45% of palmitoleic acid (C16:1), ≈30% of oleic acid (C18:1), ≈17% of palmitic acid (C16:0), and ≈5% of stearic acid (C18:0; Figure 1b). The deletion of EBP genes had little effect on the relative proportions of fatty acids. The fatty acid profile of the *erg6*Δ mutant was close to the ones of the wild type strains. For the *erg2*Δ*erg6*Δ mutant, the only slight difference consisted of a decrease of oleic acid (20%) and an increase of myristic acid (C14:0) (5%).

### 3.2. Wild-Type (WT) Yeasts Are More Resistant to Oxidative Treatment Than erg6Δ and erg2Δerg6Δ Strains 

**Figure 2:**
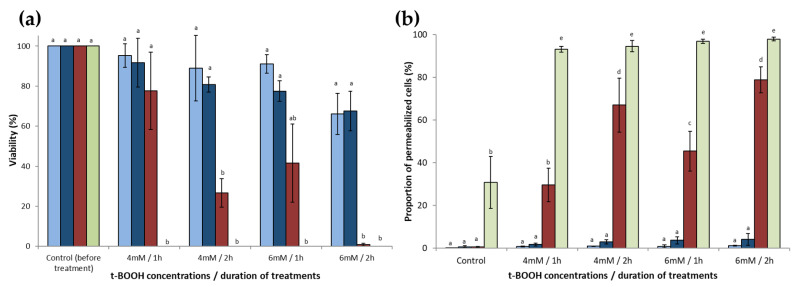
Resistance of *S. cerevisiae* to treatment with t-BOOH**.** After growth in the early stationary phase, BY4742 WT (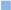), RH448 WT (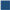), *erg6*Δ (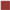), and *erg2*Δ*erg6*Δ (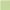) strains of *S. cerevisiae* were washed, adjusted to a concentration corresponding to DO_600 nm_ = 0.5, and placed in PBS containing t-BOOH (4 or 6 mM) for 1 or 2 h. (**a**) After treatment, the cells were washed and spotted at different ten-fold dilutions (from 10^−1^ to 10^−4^) to assess the cell viability. (**b**) Plasma membrane integrity was assessed by PI staining of cells and flow cytometry analysis. Data are presented as mean values ± standard deviation of three independent experiments. ANOVA was performed on R v3.6.1 software and if it was significant (*p* < 0.01), Tukey’s HSD (Honest Significant Difference) test was performed to observe significant differences among conditions. Letters a, b, c, d, and e represent significantly different groups.

The effect of the molecular species of sterol on yeast resistance to oxidative perturbation was assessed by cell exposition to t-BOOH, an oxidizing agent that initiates membrane lipid oxidation. The cultivability and plasma membrane integrity of both strains were measured after oxidative treatment with t-BOOH (4 or 6 mM) for 1 or 2 h (Figure 2).

The *erg2*Δ*erg6*Δ mutant was very sensitive to t-BOOH treatments. No cultivable cells were observed after the different treatments, even for the less severe one (4 mM, 1 h). The *erg6*Δ strain was also more sensitive to this treatment than the WT strains. The viability of the *erg6*Δ mutant strain was 77% and 26% after 1 and 2 h treatment, respectively, with 4 mM t-BOOH, and 41% and 0.9% after 1 and 2 h treatment, respectively, with 6 mM t-BOOH (Figure 2a). Therefore, the decrease in the viability of this strain was dependent on the concentration of t-BOOH and the contact time. The viability of the BY4742 and RH448 WT strains decreased to 66% and 68%, respectively, after the most severe treatment (6 mM, 2 h; Figure 2a). A complementary approach to assess yeast sensitivity was performed by plating yeasts onto solid growth media containing different concentrations of t-BOOH (Appendix B). The higher sensitivity of the mutant strains in comparison with the wild type strains has been confirmed. 

Plasma membrane integrity was also evaluated for both strains after treatment with t-BOOH (Figure 2b). The membrane integrity of the *erg2*Δ*erg6*Δ strain was strongly affected after t-BOOH treatments. The proportion of permeabilized cells was higher than 95% after the different treatments. We also observed that the control cells (without t-BOOH treatment) of this double mutant presented approximately 30% of permeabilized cells, indicating an initial fragility of the cells without oxidative treatments. For the *erg6*Δ strain, the proportion of permeabilized cells increased with the concentration of t-BOOH and the duration of treatment to reach 79% after 2 h treatment with 6 mM t-BOOH. The membrane integrity of the WT strains was hardly affected by any of the treatments.

The viability and membrane integrity results indicate that the decrease of yeast survival during t-BOOH treatment was mainly related to plasma membrane permeabilization. 

### 3.3. Sterols Protect Lipids from Oxidation in Liposomes 

The results obtained with the WT and EBP mutant strains suggested that ergosterol is involved in yeast resistance to oxidative perturbation. To clarify the role of ergosterol, liposomes prepared from natural polar lipid extracts of *S. cerevisiae* containing zymosterol (accumulated by the *erg6*Δ and *erg6*Δ*erg2*Δ strains), cholesta-5,7,24-trienol (accumulated by the *erg6Δ* strain), or ergosterol (accumulated by wild type strains) were subjected to oxidative perturbation. The kinetics of lipid peroxidation were assessed using the ratiometric fluorescent probe C11-BODIPY 581/591 (C11-BP) [33].

**Figure 3:**
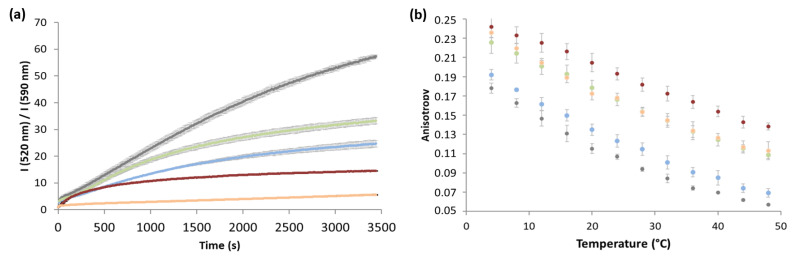
Effect of the sterol content of the liposome properties**.** Liposomes were made from yeast lipid extracts without sterol (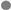), with a zymosterol/phospholipid molar ratio of 1/3 (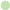), with a cholesta-5,7,24-trienol/phospholipid molar ratio of 1/3 (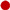), or with an ergosterol/phospholipid molar ratio of 1/3 (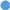). Control experiments were performed with lipososmes with a tocopherol/phospholipid molar ratio of 1/3 (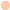). (**a**) Phospholipid oxidation induced by cumene hydroperoxide and haemin was assessed by the oxidation kinetics of BP-C11 in liposomes with different sterol compositions. Oxidation was expressed as the intensity ratio (I_520 nm_)_oxidized_/(I_590 nm_)_non-oxidized_. Oxidation was started at t = 0 s. (**b**) Fluidity of liposomes was assessed by steady-state DPH fluorescence anisotropy measurement as a function of temperature in the range 4–48 °C. The results are presented with error bars corresponding to the standard deviation calculated from three repeated experiments.

Oxidative perturbation was induced by adding cumene hydroperoxide and haemin to the liposome suspension (Figure 3a). An increase in the I_520 nm_/I_590 nm_ ratio of C11-BP fluorescence corresponded with an increase in lipid peroxidation. Five compositions of liposomes were tested, as follows: with ergosterol, cholesta-5,7,24-trienol, or zymosterol; without sterol; and with tocopherol, a well-known antioxidant that was used as a control. Liposomes prepared without sterols exhibited the fastest oxidation kinetics with a final ratio of C11-BP, close to 57 after 3400 s (Figure 3a). When zymosterol, cholesta-5,7,24-trienol, or ergosterol were added during liposome preparation, the kinetics of oxidation were slowed and the final ratios were decreased to 33.1, 14.5, and 24.5, respectively. Lipid peroxidation was drastically reduced in the liposomes containing tocopherol, with a final ratio of 5.5.

Sterols are known to differentially affect the membrane structure, packing, and fluidity. Rigidification of the lipid bilayer of liposomes by sterols could play a role in the protection of phospholipids against oxidation by reducing the accessibility and the mobility of radical species to the core of the membrane. To test this hypothesis, the fluidity and structure of the liposomes were assessed by fluorescence anisotropy after lipid bilayer staining with DPH (Figure 3b). Steady-state fluorescence anisotropy measurements were performed over a range of temperatures between 4 °C and 48 °C. Independent of the composition of the liposomes, decreasing the temperature led to a progressive increase in anisotropy, reflecting the global rigidification of the lipid bilayer without net lipid phase transition. The absence of a net lipid phase transition is probably related to the diversity of phospholipids in yeast lipid extracts: each type of phospholipid displays a specific phase transition temperature. However, the global rigidity of the liposomes depended on the presence of sterols or tocopherol. Sterols increased bilayer rigidity at a given temperature with an effect depending on the nature of sterols: cholesta-5,7,24-trienol > zymosterol > ergosterol. Tocopherol showed similar stiffening properties to zymosterol.

### 3.4. Ergosterol Displays Greater 2,2-Diphenyl-1-Picrylhydrazyl (DPPH) Radical-Scavenging Activity Than Zymosterol

**Figure 4:**
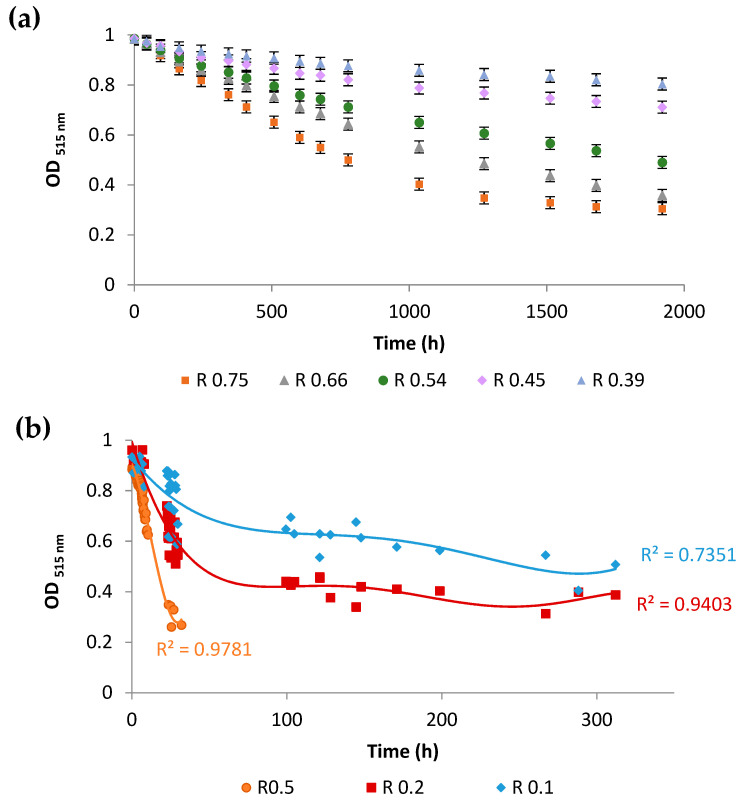
Assessment of the anti-oxidant activity of sterols using the stable free radical diphenylpicrylhydrazyl (DPPH). (**a**) Kinetics of the decrease of the absorbance at 515 nm for different zymosterol/DPPH^●^ ratios (R). Error bars represent the 95% confidence interval. (**b**) Kinetics of the decrease of the absorbance at 515 nm for different ergosterol/DPPH^●^ ratios (R). Each of the kinetics were modelled by polynomial law (30 points per kinetic). With the Pearson’s table (critical values of correlation coefficient), using a value of level of significance for a two-tailed test of 0.001, and with 30 points (freedom degree = 28), the critical r value is 0.570. The values of regression coefficients reflect a suitable fit.

The DPPH method is widely used to assess the radical-scavenging activity of molecules. This method is based on the reduction of the optical density at 515 nm, the maximum wavelength of the radical chromophore DPPH^●^. The kinetics of the radical scavenging activity of zymosterol and ergosterol are presented in Figure 4a,b, respectively. Cholesta-5,7,24-trienol, which is a very expensive molecule, was not used because the DPPH method requires huge quantities of sterols.

Nearly 33 h (around 2000 min) are necessary to exchange all radicals, with an ergosterol/DPPH ratio of 0.5, versus 2000 h (nearly 2 months) with a zymosterol/DPPH ratio of 0.54. These results clearly demonstrate that ergosterol is a much more efficient radical scavenger than zymosterol. The antioxidant activity measured by the DPPH-scavenging method is often reported as (i) EC_50_, defined as the effective concentration of the antioxidant necessary to decrease the initial DPPH concentration by 50%, and (ii) t_50_, the time needed to reach the steady state with EC_50_. Based on the kinetics at 25 different zymosterol/DPPH ratios, and after extended storage and OD recording (more than 2 months to reach a stable final value; data not shown), the EC_50_ was determined to be 5.2 × 10^−5^ ± 3 × 10^−7^ M and t_50_ to be around 1417 ± 404 h (five zymosterol/DPPH ratios are presented in Figure 4a). Using the kinetics at 30 different ergosterol/DPPH ratios (data not shown), the EC_50_ was determined to be 1.1 × 10^−5^ ± 6.4 × 10^−8^ M and t_50_ around 300 ± 17 h (three ergosterol/DPPH ratios are presented in Figure 4b). The radical scavenging efficiency (RSE) for ergosterol was much higher than that for zymosterol, with respective values of 5.1 ± 0.3 L mol^−1^min^−1^ and 0.24 ± 0.07 L mol^−1^min^−1^.

### 3.5. Computational Analysis of Antioxidant Properties of Sterols

To understand the role of sterols in the prevention of phospholipid oxidation, we must consider the possible reactions between sterols and free radicals (R**^●^**). Four reaction-type mechanisms are possible:

**(1)**  **Hydrogen Atom Transfer (HAT)**

   Sterol + R**^●^** → [Sterol_(-H)_] **^●^** + HR

**(2)**  **Electron Transfer (ET)**

   Sterol + R**^●^** → [Sterol] **^●+^** + R**^−^**

**(3)**  **Electron Transfer followed by Proton Transfer (ET-PT)**

   Sterol + R^●^ → [Sterol]**^●+^** + R**^−^** → [Sterol_(-H)_]**^●^** + HR

**(4)**  **Radical Adduct Formation (RAF)**

   Sterol + R**^●^** → [Sterol-R] **^●^**

The HAT and ET-PT mechanisms lead to the formation of the same end-products, namely free-radical sterols. However, ET-PT involves an intermediary step that corresponds to the ET reaction with the formation of a radical cation from sterol. The RAF mechanism consists of the formation of a bond between the radical and the sterol, allowing for the stabilization of the radical. In a previous thermodynamic study of ergosterol, it was shown that the Gibbs free energies of the RAF reactions were endergonic or close to zero, meaning that these reactions are unfavorable [34]. Therefore, we did not consider these reactions in our study. We first assessed the stability of the sterol radical cations and their electronic structure.

**Figure 5:**
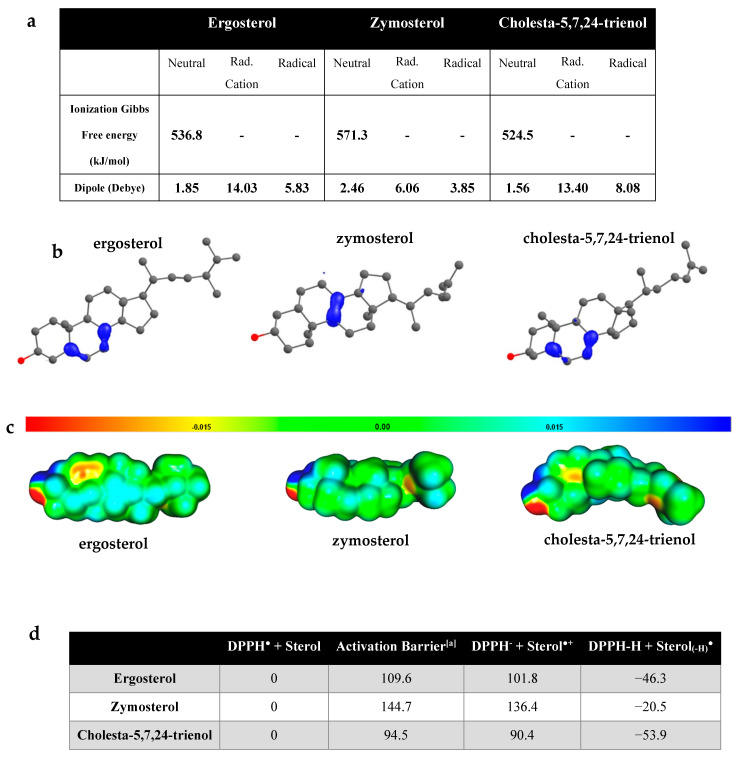
Computational study of the antioxidant properties of ergosterol, zymosterol, and cholesta-5,7,24-trienol. (**a**) B3LYP/6-311G(2d,p)-calculated properties for ergosterol, zymosterol, and cholesta-5,7,24-trienol species. (**b**) Representation of spin density of the radical cations of ergosterol, zymosterol, and cholesta-5,7,24-trienol. (**c**) Electrostatic potential maps of sterols. Blue indicates more positive regions; red indicates more negative regions. (**d**) Relative Gibbs free energies (at 298 K) in kJ/mol of the main intermediates for the reaction of DPPH radicals with ergosterol, zymosterol, and cholesta-5,7,24-trienol species. The activation barrier for the electron transfer was estimated using the Marcus theory.

The radical cations (RC) formed from neutral sterols by the loss of an electron are high-energy intermediates with ΔG values higher than 500 kJ/mol (Figure 5a). The higher stability of the ergosterol and cholesta-5,7,24-trienol radical cations with respect to the zymosterol one comes from the fact that the single electron is more delocalized on ergosterol and cholesta-5,7,24-trienol radical cations, as shown by the spin density maps of Figure 5b. This larger delocalization comes from the conjugated double bond present in ergosterol and cholesta-5,7,24-trienol, but not in zymosterol.

The computed dipoles suggest that upon oxidation, the ergosterol RC may move in the cell membrane (between the core and the surface of the membrane) because of an increased polarity between the stable molecules and their respective RCs. In addition, the higher ionization energy for the ergosterol free radical indicates a greater electronic stability of this species compared with the zymosterol free radical. It is also interesting to compare the Map of Electrostatic Potential (MEP; Figure 5c), which illustrates the presence of partial charges on the molecule. The electrostatic potential of molecules is also related to the molecule polarity. The lower electrostatic potential of zymosterol in comparison with ergosterol confirms the difference of polarity between the two sterol molecules. Radical cations are more polar than their neutral counterpart, as evidenced by their much higher dipoles (Figure 5a): this higher polarity of the ergosterol RC facilitates its displacement to a more polar region of the lipid bilayer. It is worth noting that the radical cations of ergosterol are much more polar than that of zymosterol.

We then computed the Gibbs free energy evolution for the reaction of each sterol with the DPPH radical, as gathered in Figure 5d. The ET step with DPPH is endergonic for all three sterols (Figure 5b). This indicates that the ET reaction will only occur if it is followed by the exergonic proton-transfer step. Interestingly, the relative free energies of the radical cation intermediate and the final radical followed the same order: cholesta-5,7,24-trienol < ergosterol < zymosterol. Consequently, computational results were in agreement with the fact the ergosterol reacts faster with DPPH° than zymosterol (Figure 4).

While a full mechanistic study is beyond the scope of this article, we characterized the transition state for the HAT reaction between ergosterol and the DPPH radical (see Appendix C). The activation barrier is equal to 236.4 kJ/mol, much higher than those estimated for the ET step. Therefore, the ET-PT mechanism seems to be the most plausible for the antioxidant mechanism of these sterols.

From these results, we can conclude that the characteristics obtained in the oxidation modelling of these sterols indicate that cholesta-5,7,24-trienol and ergosterol, but also to a lesser extent zymosterol, can act as antioxidants via the ET-PT mechanism. ET greatly increases the polarity of sterols and could induce the migration of sterol RC towards the surface of the lipid bilayer, a location that may favor deprotonation to complete the ET-PT mechanism.

## 4. Discussion

The conventional approach to assessing the role of sterol in yeasts is the use of mutant strains in which the genes involved in the EBP are deleted. The last five genes of this pathway are non-essential and their deletion leads to mutant strains that accumulate different sterols [32]. In this way, it was shown that ergosterol is involved in yeast resistance to chemical and physical perturbations [9,17,35,36]. In the present study, a comparison of the resistance of *erg6*Δ, *erg2*Δ*erg6*Δ, and WT strains revealed that the accumulation of zymosterol in place of other sterols, namely ergosterol, made yeasts more sensitive to oxidative perturbations induced by t-BOOH (Figure 2a), a chemical oxidant that targets membrane lipids. The high susceptibility of the mutant strains was correlated with the loss of plasma membrane integrity (Figure 2b). Peroxidation of phospholipids modifies their structural characteristics, which leads to alterations in the molecular organization of the plasma membrane. Oxidative agents react with the unsaturated acyl chains of phospholipids, leading to the formation of lipid hydroperoxides. The presence of hydroperoxides in the membrane causes an increase in water permeability and the modification of membrane order and fluidity [37,38,39]. When extensive peroxidation occurs, the formation of pores in membrane has been reported [40,41]. The degree of plasma membrane fatty acid unsaturation and the yeast sensitivity to oxidative stress are closely related [42]. The fatty acid profiles of the different strains of our study were very similar (Figure 1b), with a high content of mono-unsaturated fatty acids. Thus, the difference in the sensitivity between strains cannot be explained by the difference in the unsaturation of fatty acids. Three mechanisms could explain the higher resistance of the WT strains compared with the EBP mutant strains.

(i) The first mechanism is related to the structural effect of sterols. In contrast to the destabilizing effect of lipid peroxidation products, sterols could exert a stiffening effect on the plasma membrane. It has been reported that the addition of cholesterol to the lipid bilayer could maintain the structure of lipoperoxidized membranes based on its ability to counteract fatty acid disordering [43]. This effect could probably be related to the nature of the sterol molecule and could explain the greater destabilization of membranes during the accumulation of oxidized phospholipids in the mutants compared with the WT strains.

(ii) A second hypothetical mechanism is based on the antioxidant activity of sterols themselves. It is well known that sterols are able to be oxidized and the literature dealing with oxysterols is increasingly abundant [44]. Several authors have suggested that sterols could be the point of weakness for cells during oxidative perturbations [45,46]. However, another hypothesis proposes that the oxidation of membrane sterols could avoid the peroxidation of phospholipids. Outside the cellular context, it has been shown that the presence of sterols in phospholipid mixtures prevents phospholipid peroxidation. The protective effect of sterols depends on the nature of the sterols [47], and ergosterol could fulfill this function [19,48]. The hypothesis that sterols act as endogenous cell antioxidants has been proposed for cholesterol [49] and ergosterol [9]. However, this activity has not yet been clearly demonstrated.

(iii) Finally, the higher sensitivity of the EBP mutant strains compared with the WT could originate from the effect of *erg6* and *erg2* gene deletion on metabolic pathways or regulatory networks other than the ergosterol biosynthetic pathway. Indeed, yeasts have multiple antioxidant systems that can act to protect cells from oxidative damage [50]. Rather than being the result of an antioxidant effect of ergosterol, the better survival of the WT strains compared with the mutants could be explained by an indirect effect of the deletion of a gene in the EBP on one or more of these antioxidant systems. In a study by Gazdag and colleagues [51], it was concluded that the unbalanced redox state of an *erg* mutant (*erg5*Δ) could be at the origin of the higher sensitivity to t-BOOH of the mutant in comparison with the wild type strain. The redox state of the mutants used in our study (*erg6*Δ and *erg2*Δ*erg6*Δ) could also display this unbalance.

In our study, the addition of zymosterol, cholesta-5,7,24-trienol, or ergosterol in liposomes (prepared from natural polar lipid extracts of *S. cerevisiae*) led to a decrease in the rate of phospholipid oxidation induced by cumene hydroperoxide and haemin (Figure 3), suggesting that sterols themselves protect phospholipids. This effect was greater for cholesta-5,7,24-trienol and ergosterol than for zymosterol, without reaching the protective level of tocopherol. This result is in agreement with a previous study that showed that ergosterol inhibits iron-dependent liposomal lipid peroxidation [48]. To our knowledge, the protective effect of cholesta-5,7,24-trienol has not been studied. These in vitro liposome experiments allowed us to show the antioxidant properties of sterols in a membrane context without other cellular contributing factors. A comparison of the structural effects of the different sterols and tocopherol on liposomes showed that ergosterol slightly increases the rigidity of the lipid bilayer, whereas cholesta-5,7,24-trienol and zymosterol strongly rigidify the membrane. The rigidifying effect of zymosterol was similar to tocopherol (Figure 3b). Thus, the rigidifying effect of sterols and phospholipid protection from oxidation were not correlated. This indicates that the antioxidant property of sterols is not strictly related to their structural effects.

Beyond in vivo experiments, the antioxidant properties of molecules can be assessed by in vitro and in silico approaches. In our study, we showed that sterols display a DPPH radical scavenging activity, with a greater activity for ergosterol than for zymosterol (Figure 4). Computational studies based on quantum chemistry have been shown to be a very effective tool to provide evidence and to understand the structure–activity relationship of different compounds. This method has been widely used to explain the antioxidant properties of numerous molecules. Quantum-chemical calculations revealed that ergosterol, zymosterol, and cholesta-5,7,24-trienol could react with radical species and exhibit antioxidant properties by means of an ET-PT mechanism. In this mechanism, radical cations from sterols are first formed by the loss of an electron and are then transformed into free radicals by proton abstraction. However, zymosterol radical cation is less stable than the radical cations of ergosterol and cholesta-5,7,24-trienol (Figure 5d). This difference is based on the presence of two conjugated double bonds in the B-ring of the ergosterol and cholesta-5,7,24-trienol molecules, which allows for the delocalization of unpaired electrons over several atoms (Figure 5b), thereby conferring greater stability on the radicals compared with localized radicals. By their reaction with radical species, sterols and mainly sterols having two conjugated double bonds of the B-ring, as ergosterol and cholesta-5,7,24-trienol, could prevent phospholipid peroxidation in the lipid bilayer. A previous study showed that sterols exhibited a higher reactivity than fatty acids with a hydroxyethyl radical [52].

The basis of the specificity of sterols in each eukaryotic kingdom is intriguing. In particular, the reason for ergosterol accumulation by fungi is unclear, because its synthesis requires more energy than that of cholesterol [7], and structure−function studies did not show any benefits of ergosterol compared with cholesterol [53]. The results of our study revealed that sterols are not only structural compounds of the plasma membrane of eukaryotic cells, but can also display antioxidant properties. These are particularly remarkable for ergosterol and cholesta-5,7,24-trienol, whose structure contains two conjugated double bonds. In vivo experiments performed on yeasts revealed that the *erg2*Δ*erg6*Δ strain, for which sterols with two conjugated double bonds represented 4% of sterols (3% cholesta-5,7,24-trienol and 1% ergosta-5,7-dienol), was the most sensitive strain tested in our study (Figure 1 and Figure 2). Even if more resistant than *erg2*Δ*erg6*Δ, the *erg6*Δ strain was more sensitive than the wild type strain (Figure 2). A possible explanation for this result could be the difference in the proportion of sterols with two conjugated double bonds. Indeed, the *erg6*Δ strain presented 69% of sterols with two conjugated double bonds (23% cholesta-5,7,22,24-tetraenol and 46% cholesta-5,7,24-trienol), while the wild type strain presented 87% (77% ergosterol, 8% ergosta-5,7-dienol, and 2% ergosta-5,7,9(11),22-tetraenol; Figure 1). In the cellular context of yeasts, cholesta-5,7,24-trienol is not accumulated in such a large proportion as ergosterol is. This sterol, as with other ergosterol precursors accumulated by the EBP yeast mutants, is not mainly accumulated by environmental yeasts, which accumulate ergosterol in a large proportion. Therefore, ergosterol may be the most efficient molecule to both provide structural functions of the yeast plasma membrane and to protect it from oxidation. Its hydrophobic nature could result in its displaying scavenging activity in the vicinity of the lipids’ double bounds. A recent study suggested that ergosterol could interact preferentially with mono-unsaturated fatty acids, whereas cholesterol is able to interact with saturated lipids [54]. In such a context, the presence of ergosterol close to the unsaturated phospholipid acyl chains of yeast may constitute a key element explaining the uncommon resistance of yeast to desiccation. These results reinforce that ergosterol has been retained during evolution in late branching fungi because it serves as a consensus sterol, being able to satisfy both the structural and antioxidant functions [9]. These functions are crucial for those organisms that live in interfacial habitats where they experience hydric and oxidative perturbations. All of these knowledge elements support the Brown and Galea hypothesis, which postulates that sterols are an adaptation to aerobic life [55,56].

The results of our study strongly suggest that ergosterol is an antioxidant molecule that prevents phospholipid oxidation in the cellular context. This role has rarely been associated with sterols, and it will be interesting to study this function in cholesterol and phytosterols. In eukaryotic cells, sterols are not homogeneously distributed in the plasma membrane, but enrich specific membrane domains. In mammalian and plant cells, these domains, called lipid rafts, are very small (not visualizable by optical microscopy) and transitory in time [57]. The plasma membrane of yeasts contains large and stable domains called microdomains, among which some are enriched in ergosterol [58]. These ergosterol-rich microdomains could be hypothetically considered as antioxidant islets in the yeast plasma membrane. Moreover, a study focusing on yeast plasma membrane organization showed that ergosterol is excluded from sphingolipid-enriched gel domains [59]. In this model, ergosterol is surrounded by unsaturated phospholipids. Thus, the location of ergosterol in the vicinity of acyl chain unsaturation could be particularly favorable for preventing phospholipid oxidation. Future investigations should take into account the antioxidant properties of sterols to clarify their roles in their local context, i.e., the hydrophobic core of the phospholipid bilayer and cell membrane domains.

## 5. Conclusions

By using in vitro and in silico approaches, we showed in our study that ergosterol acts as an antioxidant molecule that could be involved in yeast resistance to oxidation. In the cellular context, the antioxidant function of ergosterol could be of prime importance for yeasts and other fungi to resist oxidative stress. Specifically, free ergosterol accumulated in the plasma membrane of fungi cells could allow for the protection of phospholipids against oxidative perturbations. Thanks to a computational study based on quantum chemistry, we demonstrated that the antioxidant ability of ergosterol operates through an electron transfer, followed by proton transfer mechanism, and is related to the presence of the two conjugated double bonds of the B-ring of the sterol structure. Outside the cellular context, ergosterol might be used as a natural antioxidant molecule to prevent the oxidative degradation of foods. Finally, the antioxidant function of the ergosterol molecule could play a role in the incompletely understood beneficial effects of some mushrooms on health.

## Figures and Tables

**Figure A1:**
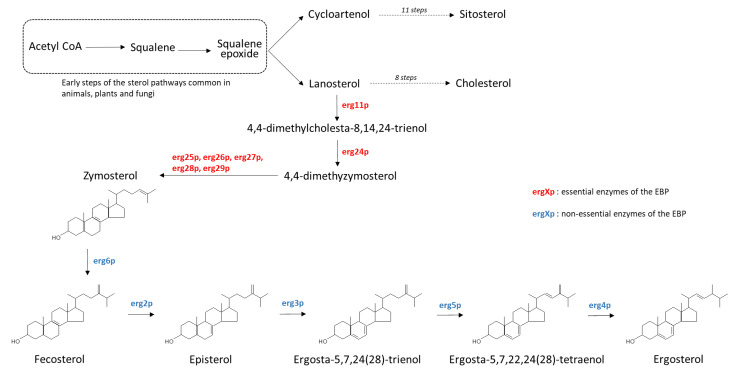
Schematic representation of the ergosterol biosynthesis pathway from acetyl CoA. Plants, animals, and fungi have a common early sterol synthetic pathway up to squalene epoxide. After this compound, cycloartenol is produced in plants, whereas squalene epoxide gives lanosterol in animals and fungi. The end-products of the sterol pathways are sitosterol, cholesterol, and ergosterol for plants, animals, and fungi, respectively. The final enzymes involved in the ergosterol biosynthesis (ergXp) are described.

**Figure A2:**
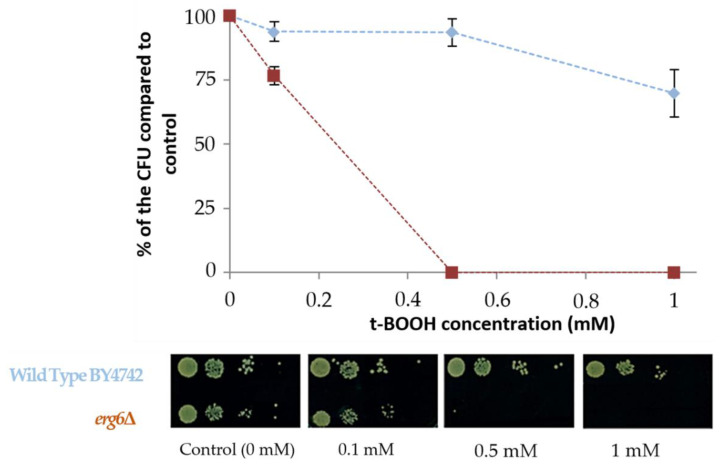
Tolerance of *S. cerevisiae* to various concentrations of t-BOOH in the growth medium. WT BY4742 (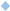) and *erg6*Δ (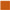) strains of *S. cerevisiae* are grown for 48 h at 25 °C in a yeast extract–peptone–dextrose (YPD) medium supplemented with various concentrations of t-BOOH (0, 0.1, 0.5, or 1 mM). Cells were spotted at different ten-fold dilutions (from 10^−1^ to 10^−4^) from the initial suspension at OD_600 nm_ = 0.5. Data are presented as mean values ± standard deviation of three independent experiments.

## Data Availability

Data are contained within the article.

## Appendix B

Complementary to the experiment consisting in placing yeasts in contact of t-BOOH in PBS medium, yeast resistance to oxidation was assessed by plating a yeast suspension on solid growth YPD medium containing low concentrations of tert-butyl hydroperoxide (t-BOOH; 0.1, 0.5, or 1 mM) and the growth of the strains compared after 48 h. Because of the very high sensitivity of the *erg2*Δ*erg6*Δ double mutant, no growth was observed regardless of the t-BOOH concentration (data not shown). WT BY 4742 strain growth was weakly affected by the presence of t-BOOH: at the highest concentration used (1 mM), the number of colony-forming units (CFU) was 70% of that in the control (without t-BOOH). In contrast, the growth of the *erg6*Δ strain decreased dramatically with increasing concentrations of t-BOOH and reached 0.008% of the control at a concentration of 0.5 mM. No growth was observed at 1 mM.

## Appendix C

### Appendix C.1. Estimation of the Electron Transfer Activation Free Energy

Electron transfers are usually described using collective reaction coordinates, as first described by Marcus [60]. In the Marcus theory, the activation free energy is as follows:

$$\Delta_{r}G^{‡}=\frac{\lambda }{4}{\left(1+\frac{\Delta G_{ET}}{\lambda }\right)}^{2}$$

$\Delta G_{ET}$ is the Gibbs free energy of electron transfer (*ET*) reaction and λ is the reorganization energy. The reorganization energy was calculated as

$$\lambda =\Delta E_{ET}-\Delta G_{ET}$$

where $\Delta E_{ET}$ is the nonadiabatic energy difference between the reactants (DPPH^●^+ sterol) and the vertical products (i.e., DPPH^−^ + Sterol^●+^ in the same geometry as the reactants) [61].

### Appendix C.2. Bond Dissociation Energies (kJ/mol) for Several H Atoms

By inspecting the spin density (with a low iso-value of 0.004), it appeared that many CH bonds were polarized and could thus lead to the formation of different radical species. We thus computed the bond dissociation energies (BDE) of these bonds to obtain the relative stabilities of the possible radical molecules: the most stable radical corresponds to the lowest BDE (show in green).
